# Cancer Biomarkers from Genome-Scale DNA Methylation: Comparison of Evolutionary and Semantic Analysis Methods

**DOI:** 10.3390/microarrays4040647

**Published:** 2015-11-27

**Authors:** Ioannis Valavanis, Eleftherios Pilalis, Panagiotis Georgiadis, Soterios Kyrtopoulos, Aristotelis Chatziioannou

**Affiliations:** National Hellenic Research Foundation, Institute of Biology, Medicinal Chemistry and Biotechnology, 48 Vassileos Constantinou Avenue, 11635 Athens, Greece; E-Mails: ivalavan@eie.gr (I.V.); epilalis@eie.gr (E.P.); panosg@eie.gr (P.G.); skyrt@eie.gr (S.K.)

**Keywords:** DNA methylation, breast cancer, B-cell lymphoma, epigenetic biomarker, classification, graph-theory, evolutionary algorithm, gene ontology tree

## Abstract

DNA methylation profiling exploits microarray technologies, thus yielding a wealth of high-volume data. Here, an intelligent framework is applied, encompassing epidemiological genome-scale DNA methylation data produced from the Illumina’s Infinium Human Methylation 450K Bead Chip platform, in an effort to correlate interesting methylation patterns with cancer predisposition and, in particular, breast cancer and B-cell lymphoma. Feature selection and classification are employed in order to select, from an initial set of ~480,000 methylation measurements at CpG sites, predictive cancer epigenetic biomarkers and assess their classification power for discriminating healthy *versus* cancer related classes. Feature selection exploits evolutionary algorithms or a graph-theoretic methodology which makes use of the semantics information included in the Gene Ontology (GO) tree. The selected features, corresponding to methylation of CpG sites, attained moderate-to-high classification accuracies when imported to a series of classifiers evaluated by resampling or blindfold validation. The semantics-driven selection revealed sets of CpG sites performing similarly with evolutionary selection in the classification tasks. However, gene enrichment and pathway analysis showed that it additionally provides more descriptive sets of GO terms and KEGG pathways regarding the cancer phenotypes studied here. Results support the expediency of this methodology regarding its application in epidemiological studies.

## 1. Introduction

Epigenetic events comprise heritable modifications that regulate gene expression without altering the DNA sequence itself and can serve as regulatory mechanisms for a wide range of biological processes [1,2,3]. DNA methylation, one of the most common epigenetic effects that take place in the mammalian genome, is actually a chemical modification, resulting in the addition of a methyl (CH3) group at the carbon 5 position of the cytosine ring. Most cytosine methylation occurs in the sequence context 5′CG3′, also called the CpG dinucleotide, and human genome contains regions of unmethylated segments interspersed by methylated regions [4].

The first epigenetic change in human tumors -global genomic DNA hypomethylation- was reported way back in the early 1980s, at about the same time the first genetic mutation in an oncogene was discovered [5,6]. Cancer was the first group of diseases considered for DNAmethylation-targeted therapeutics [7] and DNA methylation is now implicated as a critical determinant in carcinogenesis, thus becoming a topic of intense investigation the recent years [8]. Specifically, hypermethylation of CpG islands, typically a sequence of 300–3000 base pairs in length within or near to approximately 40% of promoters [9], has been related to most types of cancer including solid tumors, e.g., breast, colon, lung, or hematologic forms, e.g. leukemias [4,10,11,12,13]). Widely epigenetic effects on gene expression and proteins involved in cancer manifestation are the hypermethylation of tumor suppressor genes and the aberrant expression of DNMT genes [14]. In the other hand, hypomethylation is gaining importance, as it has been observed frequently in solid tumors [15], such as hepatocellular and prostate cancers, and can be attributed to two forms. Firstly, hypomethylation of transcription regulatory regions can take place (e.g. see [16] for the prognostic value of demethylation of urokinase promoter in patients with breast carcinoma), however this is much less frequent than hypermethylation of CpG islands overlapping promoters [17]. Secondly, global DNA hypomethylation is observed frequently in cancers, e.g. hypomethylation of DNA cancer-hypomethylatedrepeats of tandem centromeric satellite α, juxtacentromeric (centromere-adjacent) satellite 2, interspersed Alu, and long interspersed elements repeats [17,18,19]. Hypomethylation of DNA is generally more pronounced with tumor progression or the degree of malignancy [13,20].

DNA methylation in cancer cells may be modified by a number of factors like aging of tissues, nutrition, and environment [21,22,23]. Recently, the rapid progress in microarray technologies has opened new avenues for the high-throughput monitoring of epigenetic effects [24,25]. One of the latest microarray platforms is the Illumina’s Infinium Human Methylation 450K Bead Chip, which can detect CpG methylation changes in more than 480,000 cytosines distributed over the whole genome [26]. The nature of DNA methylation data imposes serious restrictions concerning the successful porting of popular statistical tools and methodologies developed for transcriptomic analysis. Thus, pre-processing and analysis of targeted bisulfite sequencing microarrays is a challenging research area where no gold-standard methods have been proposed.

From an artificial intelligence perspective, the identification of biomarkers is considered a complex task, where feature selection is indispensable prior to the classification task, which separates various physiological states (*i.e.*, disease stages) [27,28]. The derivation of a small feature set that best explains the difference between the biological states is aiming to yield robust, well-performing classifiers and renders the problem computationally tractable. Feature selection represents, in general, a prerequisite for the setup of reliable classification models in the area of bioinformatics, given the usually high dimensionality of the feature spaces observed in microarray analyses [29]. While feature selection and classification methods have been comprehensively explored in the context of gene expression data, little work has been done on how to perform feature selection or classification in the context of epigenetic data. Given the importance of epigenomics in cancer and other complex genetic diseases, it is critical to identify the appropriate statistical methods to be used in this novel context. So far, related studies have focused on the derivation of biomarkers using cell lines or the 27K DNA Methylation Array by Illumina [30,31,32].

In the current study, we employ a data mining framework for the analysis of genome-scale epigenetic data that have been produced by Illumina’s Infinium Human Methylation 450K Bead Chip [26]. We aim to examine, retrospectively, the manifestation of two cancer types (breast cancer and B-cell lymphoma) through examination of genome-scale measurements of the DNA methylation observed in blood samples, collected from an Italian epidemiological cohort. At the time of collection all donors were considered healthy. In this sense, the aim is to discover retrospectively early molecular predictive markers of disease, probably years before its macroscopic observation, by correctly classifying samples in three disease related categories. These correspond to healthy, breast cancer, and B-cell lymphoma categories which, phenotypically, are considered essentially disparate and at the same time are extremely broad, encompassing very diverse combinations of molecular phenotypes (groups of genes or other molecules orchestrating disease emergence and progression). For our purposes, we have developed a workflow consisting of a feature selection module, three-class (control *vs.* two cancer types) or two-class (control *vs.* either cancer type, one cancer type *vs.* another) classification modules. Feature selection is based on two different methodologies: (i) an evolutionary algorithm, which belongs to the class of meta-heuristic optimization methods inspired by biological evolution and (ii) the GORevenge algorithm, a graph-theoretic methodology, published previously by [33], which exploits semantics, *i.e.* data represented on structured knowledge models like ontologies, included in the Gene Ontology (GO) tree. It is the first time to the authors’ knowledge that an artificial intelligence based pipeline is applied to the extended version of Illumina Bead Chip arrays.

Data came from an Italian epidemiological cohort consisting of samples organized in control, breast cancer, and B-cell lympoma classes. The available samples have been randomly split into two independent datasets: (i) a training set used for feature selection, training various popular classifiers and their evaluation through resampling and (ii) a testing set, which consists of samples that have not been involved at all in the training of the classifiers and is used as an independent set for the application of a real-world evaluation scheme. The pre-processing methodology, previously presented by authors in [34], includes: (i) the correction of the methylation signals, using a novel intensity-based correction method and appropriate quality controls, and (ii) a statistical pre-selection of candidate CpG sites to be used for our data mining purposes in the current study. Data are analyzed through Rapidminer, a freely available open-source data mining platform that integrates fully the machine learning WEKA library, and additionally process and usage of data and metadata [35]. Results show that subsets of features, corresponding to CpG sites, delivered by the feature selection modules could represent predictive biomarkers for the two cancer types studied. Furthermore, encouraging classification performance measurements could be obtained by the series of classifiers. Gene enrichment and pathway analysis which followed evaluated the biological content of the subsets of CpG sites delivered by the two selection methods.

## 2. Experimental Section

### 2.1. Cohorts and Samples

The study was conducted in the context of the European EnviroGenoMarkers project (Available online: www.envirogenomarkers.net) and involved individuals, from the European Prospective Investigation into Cancer and Nutrition study (EPIC-ITALY). DNA extraction from buffy coats, CpG methylation profiling (using the Illumina Infinium Human Methylation 450K platform, see Section 2.2) and the corresponding data quality assessment and preprocessing, were conducted as described previously [36]. In order to address unwanted technical variation in DNA methylation analysis, normalization was carried out in two successive steps of intensity-based correction (within-chip, followed by across-all-probes) as previously described ([34], see Section 2.2, as well) making use of the DNA methylation measured in multiple replicates of a technical quality control sample distributed among the study samples. The available Italian cancer dataset encompassed 261 samples, which correspond to 131 controls, 48 breast cancer cases (BCCA), and 82 Β-cell lymphoma cases (LYCA). The samples have originated from two separate experimental studies of matched control and case samples that aimed to study epigenetics effects towards lymphoma and breast cancer onset (See Table 1 for distribution of samples into the two cohorts). The dataset for breast cancer cohort has been deposited in NCBI’s Gene Expression Omnibus [37] and is accessible through GEO Series accession number GSE52635 (Available online: http://www.ncbi.nlm.nih.gov/geo/query/acc.cgi?acc=GSE52635). The study related to lymphoma provided a wealthier set of case samples. The primary classification to be studied here was the three-class (control *vs.* two cancer types). For this task, control samples have actually originated from the two different control samples subsets available for the separate experiments for lymphoma and breast cancer. The original control samples have been, thus, unified in a wider control sample set appropriate for the three-class problem studied. Actually, from the statistical point of view the broader size of the control class aids both the statistical power and the reliability of the analysis, as it maps to a much broader set of phenotypes, that correspond to the highly subjective, even with the medical expert’s standards, definition of the healthy person. For the three-class problem, the total of 261 samples organized in Controls, BCCA and LYCA were randomly spitted into two distinct sets: (i) training set (50 Controls, 35 BCCA, 46 LYCA) used for feature selection, training various classifiers and their evaluation through resampling and (ii) independent testing set (81 Controls, 13 BCCA, 36 LYCA) used for blind testing of classifiers. The sizes of the training set was the result of trying both to keep half of the samples in the training set (the rest for testing set) and forming a balanced occurrence of the internal classes, as well as a balanced occurrence of the two types of control samples originating from the separate studies. Balanced occurrence of samples in training set would allow a properly balanced set for training successfully the classifiers. After constructing the training set, remaining samples of all three classes were allocated to the testing set. Regarding the two class problems the subsets of samples in training and test sets were randomly drawn from the corresponding sets that formed for the three-class problem (control samples are now not unified, but separated as originated from the two separated studies). For the task control *vs.* LYCA, the training set included 28 controls and 46 cases, and the testing set included 55 controls and 36 cases. For the task control *vs.* BCCA, the training set included 22 controls and 35 cases, and the testing test included 26 controls and 13 cases. For the task BCCA *vs.* LYCA, the training set included 35 BCCA samples and 46 LYCA samples, and the testing test included 13 BCCA samples and 36 LYCA samples.

**Table 1:** Distribution of samples in the cohorts used in the study.

Cohort	Control	Cases	Total
Breast Cancer Cohort	48	48	96
Β-cell Lymphoma Cohort	83	82	165
Both Cohorts	131	130	261

### 2.2. DNA Methylation Measurements and Pre-Processing

The dataset contains methylation data extracted using the more recent chip of Illumina, *i.e.*, the Infinium Human Methylation 450K BeadChip, that includes 485,577 probes (482,421 CpG sites, 3091 non-CpG sites and 65 random SNPs) (see Table 2 for the genomic distribution of CpGs classified in different groups: promoter, body, 3′UTR and intergenic [26]). Methylation of each CpG site in this chip is measured based on two channel intensities I_Meth_ and I_Un-Meth_, available for all probes, similarly with the two-channel arrays in transcriptome analysis. To date, two methods are used to measure DNA methylation. The first one is *β*-value, which is used to measure the percentage of methylation (ranging from 0 to 1). It is defined as *β* = I_Meth_/(I_Meth_ + I_Un-Meth_). *β*-value, possesses an intuitively direct, biological interpretation, expressing roughly the amount of CpG methylation measured in the collected DNA extracted from a biological sample (population of cells), as percentage of methylation. The second method applied is the widely known from its use in gene expression microarray analysis, *M*-value [38]. The logarithmic ratio of the methylated *versus* the unmethylated signal intensities, quoted as *M*-value, *M* = log(I_Meth_/I_Un-Meth_), is used by this method as a DNA methylation measurement estimate. The *M*-value, used here to measure methylation of CpG sites, expresses the part of the given, probed epigenomic region, which has evaded methylation. It is also statistically more valid and applicable in differential and other statistical analyses compared to the alternative methylation metric of *β*-value, as it is approximately homoscedastic [38].

In agreement with the well-established statistical strategy for the adoption of transformations that render the data distributions symmetric, that is widely applied in the processing of microarray data, M-values were used to measure and correct DNA methylation signal intensities. This has been done using a novel intensity based method previously presented by authors in [34], which additionally uses quality controls (QCs), corresponding to the same technical control sample embedded in the methylation arrays. M-signal distribution is normalized, taking into account the average intensity level of both channels *I* = 0.5 × log(I_Meth_ × I_Un-Meth_) and the variation of QCs incorporated in each chip which are used to estimate error estimates.

**Table 2:** Genomic distribution of CpGs classified in different groups: promoter, body, 3′UTR and intergenic [26].

CpG Location	CpGs	Subgroup	CpGs
Promoter	200,339	TSS200	62,625
		TSS1500	77,379
		5′UTR	49,525
		1stExon	10,810
Body	150,212		
3′UTR	15,383		
Intergenic	119,830		

The normalization takes place in two successive steps: (i) within-chip; and (ii) across all probes. Normalization within chip incorporates the calculation of an error estimator across all intensity levels after partitioning the intensity space I into percentiles. The probe estimates for all arrays are then updated: the corrected *M*-value of a probe results by subtracting the respective error calculated for the corresponding intensity level. Normalization across all probes is applied next exploiting the standard deviation of the *M*-values for each probe across all QCs. The *M*-values of each probe across all samples are then updated, by subtracting the probe based error estimate. In whole, the impact of technical bias in the signal estimates is mitigated through the consecutive normalization steps as already shown in [34].

### 2.3. Statistical Pre-Selection of CpG Sites

Two statistical components have been used to pre-select a wide subset of candidate biomarkers prior to applying the data mining framework presented next. Statistics were applied for two separate experiments: controls *vs.* BCCA or controls [39] *vs.* LYCA. The goal was to pre-select a subset of CpG sites, in order to further feed the data-mining framework. These actually correspond to differentially-methylated CpG sites, with a statistical significance and beyond technical variation, as extracted using the statistical components for each of the separate experiments. These components correspond to (i) a scaled coefficient variation (Scaled CV) measurement and (ii) *p*-value measurements extracted by *t*-test and corrected by bootstrap. Scaled CV represents a robust measure of the real inter-class variability observed for a probe in the whole sample pool (controlsUcases), when compared to that observed among quality control samples, which measures solely the technical variation. The greater scaled CV is, the greater the real differential methylation is (beyond technical signal variation) and more reliable is the CpG site, as a candidate differentially methylated CpG site between the two sample categories (controls *vs.* cases).

The bootstrap corrected *p*-value measurements originate from a typical paired *t*-test for extracting statistically significant differentially-methylated probes (controls *vs.* BCCA or controls *vs.* LYCA). A paired *t*-test was possible since cases have been matched with controls for each of the experiment in terms of certain characteristics, e.g., age, sex, body mass index, and pre-post menopause for the breast cancer samples and their controls. The classical statistical test is followed, however, by a bootstrap *p*-value correction that immunizes statistical findings, against the detrimental effect of multiple hypothesis bias. The idea beneath this *p*-value correction is to examine whether the *p*-values obtained from the statistical test are indeed that extreme, or they could represent random false selections (see [34]).

### 2.4. Methods for CpG sites Selection and Classification

Prior to the application of our feature selection and classification methodology, M-value correction and the two statistical components described above, were applied for the two separate experiments: controls *vs.* BCCA and controls *vs.* LYCA. For each of the experiment, the top 1% criterion in paired *t*-test bootstrap corrected p-values and a Scaled CV>1 criterion were combined in order to derive a finally-selected robust subset of CpG sites that are both significantly differentially-methylated and immunized against technical variation. Unifying the two selected robust subsets of CpG sites yielded a total of 5719 CpG sites (~1.2% of the genome-scale methylation array) and methylation measurements for these CpG sites comprise the feature vectors used in this study.

In order to select the most important features (CpG sites), corresponding to candidate epigenetic biomarkers and test their relevance in terms of accuracy they can provide when fed to popular classifiers, an appropriate workflow was built using the Rapidminer platform (Available online: www.rapid-i.com). An evolutionary feature selection algorithm within this workflow uses the training set and an embedded *k*-nn classifier, and was initially applied in order to select the most robust features, *i.e.*, CpG sites, that distinguish the three samples types. Alternatively, CpG selection was performed using the GORevenge algorithm [33], that on a functional analysis basis selects genes (consequently CpG sites for our purposes), that possess an important role in the mechanism beneath the diseases studied in terms of centrality in the GO tree. Then, various classification modules, where the selected features were imported, *i.e.* *k*-nn (*k*-nearest neighbor) classifiers, a decision tree, and a feed-forward artificial neural network, were constructed and evaluated using the training set and leave-one-out resampling. Finally, all classifiers were tested using the totally unknown testing set. In the following, the feature selection methods (evolutionary selection or GORevenge-based selection) and the classifiers used are described.

#### 2.4.1. Evolutionary Selection of CpG Sites

This feature selection method uses a genetic algorithm (GA) [40] to select the most robust features that feed an internally used, 12-nn weighted classifier [41]. GA mimics the mechanisms of natural evolution and applies biologically inspired operators on a randomly chosen initial population of candidate solutions, in order to optimize a fitness criterion. In the current study, an initial population of 500 random chromosomes is created during the first step of the GA. Each chromosome, representing a candidate solution for the feature selection task, is a binary mask of N binary digits (0 or 1), equal in number to the dimensionality of the complete features set, that reflect the use (or not) of the corresponding feature. Each chromosome is restricted to be a binary mask, corresponding to a solution that includes a subset of features, which number lies in a predefined range. The genetic operators of selection, crossover and mutation are then applied to the initial generation of chromosomes. The selection operator selects, through a roulette wheel scheme, the chromosomes to participate in the operators of crossover and mutation, based on the fitness value of each chromosome previously calculated, *i.e.*, the total accuracy (number of samples correctly classified) that the corresponding feature subset yields using the 12-nn weighted classifier on a three-fold cross validation basis. The crossover operator mates random pairs of the selected chromosomes with probability *P_c_*=0.5 based on a uniform crossover operator, while bits within a chromosome are mutated (switched from 0 to 1 or vice-versa) with probability *P_m_* = 1/N when the mutation operator is applied. The whole procedure is repeated until the stopping criterion of attaining a maximum number of generations is reached and the best performing chromosome in the last generation is the one that represents the finally chosen feature subset. The *k*-nn weighted classifier was used internally in the evolutionary algorithm due to the rather low computational cost it raises, compared to other alternatives e.g., artificial neural network, and the need for executing and evaluating the classifier for a large number of rounds within feature selection algorithm. The setting *k* = 12 comes for that the least number of samples in the testing sets used is 13, so we would like the classifier (optimized here and applied later on the testing sets) not to examine a neighbor of samples greater in size than that of one class.

#### 2.4.2. GORevenge-based Selection of CpG Sites

As we aimed at the application of an orthogonal to the evolutionary feature selection methodology, we incorporated to our study the selection of CpG sites from the pool of genes corresponding to the initially pre-selected CpG sites, exploiting their putative functional role through GORevenge (Available online: www.grissom.gr/gorevenge, [33]). Starting from a list of genes/gene ontology (GO) terms, GORevenge exploits the functional information included in the GO tree semantics and outputs a series of functionally related genes/GO terms. The finally selected genes/GO terms may be possibly not included in the inputted list; thus, it can aid the elucidation of hidden functional regulatory effects among genes and can therefore promote a system’s level interpretation.

GORevenge uses a stepwise mechanism, starting from the initially considered genes set or GO terms. In the first phase, genes are collected, not only when linked to a given GO term, but also to its neighboring ones, *i.e.*, its parents and children GO terms. These genes are considered to belong to the same functional clique, which is defined by the use of distance based functional similarity criteria. For genes that are annotated by several terms, a pruning phase follows, where GO terms are eliminated when the in-between distance of those terms falls under certain similarity distance. GOrevenge incorporates Resnik semantic similarity metrics [42] and is able to probe specific categories of the GO, *i.e.*, molecular function (MF), biological process (BP), and cellular component (CC). Finally, a prioritized list of genes is exported based on the GOs linked to them, after the pruning stage, thus measuring the centrality of the genes in the functional mechanism as proposed by initial genes/GOs.

GORevenge was applied here, using as input to the algorithm, the set of unique genes related to the pre-selected in terms of statistics CpG sites. Specifically, a set of unique 3415 genes ids were derived based in the pre-selected 5719 CpG sites and the annotation of the Infinium Human Methylation 450K BeadChip. We applied GORevenge using as input the set of 3415 genes. Resnik semantic similarity metric, Bubble genes algorithm, and a relaxation equal to 0.15 were used as algorithm parameters (see [33] for more details on the parameters). We retrieved 235 and 210 genes included in the list of genes submitted, using BP and MF functional aspects in the algorithm, respectively. The resulting genes correspond to a total of 249 unique gene ids, which in turn correspond to a total of 352 CpG sites based on the annotation of the chip. These features represent a small, conclusive set of simultaneously significantly differentiated and with high regulatory impact (they are linked with the highest number of distinct, highly non-overlapping, biological processes) genes, which may reliably be used for the training of predictive classifiers.

#### 2.4.3. Classification

Following the selection of CpG sites (used as features from now on), classification is performed to construct classifiers fed by the selected features and measure their performance, thus validating the relevance of the selected features. Three nearest-neighbor classifiers (*k*-nn, *k* = 1, 6, 12) with weights, a classification tree (the GINI index was used as a split criterion), and a feed-forward artificial neural network (ANN) of one hidden layer were used. All classification algorithms, except ANN, are described in more detail in [28]. The ANN used here was trained using the back-propagation algorithm for 1000 epochs with a learning rate equal to 0.3 and momentum equal to 0.2 that were found to be the best choices on a trial and error basis. The hidden layer used a sigmoid activation function and contained ((num. of features+num. of classes)/2 + 1) nodes. Classifiers’ performance, in terms of total accuracy (number of samples correctly classified), and class sensitivity (number of true positives in a class that were correctly classified in this class) was measured using a training set and leave-one-out resampling (tabular results presented in Supplementary Material). The same classification algorithms were evaluated, utilizing the totally unknown-independent testing set: classifiers were constructed once using the training set and applied to the samples belonging to the testing set (results presented here in tabular format and bar plots).

## 3. Results and Discussion

Starting from the list of 5719 pre-selected CpG sites, we applied the evolutionary feature selection and/or GORevenge-based selection under the following four scenarios:

(1) Keep the dimensionality of selected features relatively low, but simultaneously with a high, robust, predictive efficiency. This aspect sets a grand challenge, as the effect of the epigenetic imprinting, especially in blood tissue (a heterogeneous collection of multiple cell types of different origins), is an extremely fuzzy one, with an intricate, indirect, accumulative mechanism of action, with respect to the prospective macroscopic biological outcome, which might take place many decades afterwards. We applied the evolutionary feature selection to the list of 5719 CpG sites with a dimensionality threshold equal to 150 for all the classification schemes, *i.e.*, controls *vs.* BCCA *vs.* LYCA (142 features were found), controls *vs.* BCCA (129 features), controls *vs.* LYCA (143 features), and BCCA *vs.* LYCA (146 features). The derived subset of features were fed to the classifiers and tested either using a training set (leave-one-out resampling) or a totally unknown testing set. Results using leave-one-out resampling are presented in the Supplementary Material (Tables S1–S4). Results for the four classification schemes mentioned above on the independent testing set are presented here in Table 3, Table 4, Table 5 and Table 6, respectively.

**Table 3:** Pre-selection followed by evolutionary feature selection (up to 150 CpG sites) for the three-class problem (controls *vs.* BCCA *vs.* LYCA) (142 selected CpG sites). Results using the independent set.

Independent Set	1-nn	6-nn	12-nn	Tree	ANN
Total Accuracy	47.69	47.69	45.38	32.13	60.00
Controls Sensitivity	54.32	55.56	54.32	40.74	69.14
BCCA Sensitivity	53.85	46.15	38.46	23.08	61.54
LYCA Sensitivity	30.56	30.56	27.78	16.67	38.89

**Table 4:** Pre-selection followed by evolutionary selection (up 150 CpG sites) for the two-class problem (controls *vs.* BCCA) (129 selected CpG sites). Results using the independent set.

Independent Set	1-nn	6-nn	12-nn	Tree	ANN
Total Accuracy	64.1	71.79	69.23	53.85	76.92
Controls Sensitivity	65.38	76.92	69.23	38.46	76.92
BCCA Sensitivity	61.54	61.54	69.23	84.62	76.92

**Table 5:** Pre-selection followed by evolutionary selection (up 150 CpG sites) for the two-class problem (controls *vs.* LYCA) (143 selected CpG sites). Results using the independent set.

Independent Set	1-nn	6-nn	12-nn	Tree	ANN
Total Accuracy	52.75	59.34	54.95	53.85	64.84
Controls Sensitivity	43.64	50.91	41.82	47.27	72.73
BCCA Sensitivity	66.67	72.22	75	63.89	52.78

**Table 6:** Pre-selection followed by evolutionary selection (up 150 CpG sites) for the two-class problem (BCCA *vs.* LYCA) (146 selected CpG sites). Results using the independent set.

Independent Set	1-nn	6-nn	12-nn	Tree	ANN
Total Accuracy	97.96	93.88	93.88	93.88	97.96
BCCA Sensitivity	100	84.62	84.62	100	100
LYCA Sensitivity	97.22	97.22	97.22	91.67	97.22

(2) Keep CpG sites as proposed by GORevenge-based selection. The 352 corresponding features were fed to the classifiers constructed for the three class problem (controls *vs.* BCCA *vs.* LYCA) and tested either using the training set (leave-one-out resampling, See Table S5) or the independent testing set (Table 7). Out of the 352 CpG sites, 183 were found to be pre-selected by the statistics using the separate experiment control *vs.* BCCA and were used in the corresponding two-class problem (results in Table S6 and Table 8, for resampling and use of the independent set, respectively). 35 CpG sites were found to be pre-selected by the statistics using the separate experiment control *vs.* LYCA and were the used in the corresponding two-class problem (Results in Table S7 and Table 9).

**Table 7:** Pre-selection followed by GORevenge for the three-class problem (controls *vs.* BCCA *vs.* LYCA) (352 selected CpG sites). Results using the independent set.

Independent Set	1-nn	6-nn	12-nn	Tree	ANN
Total Accuracy	48.46	44.62	47.69	41.54	65.38
Controls Sensitivity	48.15	35.80	40.74	37.04	70.37
BCCA Sensitivity	46.15	38.46	38.46	38.46	69.23
LYCA Sensitivity	50.00	66.67	66.76	52.78	52.78

**Table 8:** Pre-selection followed by GoRevenge for the two-class problem (controls *vs.* BCCA) (183 selected CpG sites). Results using the independent set.

Independent Set	1-nn	6-nn	12-nn	Tree	ANN
Total Accuracy	84.62	82.05	74.36	48.72	84.62
Controls Sensitivity	84.62	84.62	84.62	42.31	84.62
BCCA Sensitivity	84.62	76.92	53.85	61.54	84.62

**Table 9:** Pre-selection followed by GoRevenge for the two-class problem (controls *vs.* LYCA) (35 selected CpG sites). Results using the independent set.

Independent Set	1-nn	6-nn	12-nn	Tree	ANN
Total Accuracy	53.85	54.95	54.95	53.85	54.95
Controls Sensitivity	41.82	40	34.55	52.73	61.82
LYCA Sensitivity	72.22	77.78	86.11	55.56	44.44

(3) Perform evolutionary feature selection to the 352 CpG sites proposed by GORevenge. We applied the evolutionary feature selection with a dimensionality threshold equal to 150 for the three class problem (controls *vs.* BCCA *vs.* LYCA) and derived a subset of 141 features. These features when fed to the classifiers and tested using the training set (leave-one-out resampling) or the totally unknown testing set. Classifiers performed as reported in Table S8 and Table 10.

(4) Perform evolutionary feature selection to obtain a list of CpG sites almost equal in dimensionality to the 352 CpG sites proposed by GORevenge. We applied the evolutionary feature selection for the three-class problem (controls *vs.* BCCA *vs.* LYCA) to the list of 5719 CpG sites with a dimensionality threshold equal to 400. We derived a subset of 373 features, which were fed to the classifiers and tested using training set (leave-one-out resampling, Table S9) or the totally unknown independent testing set (Table 11).

**Table 10:** Pre-selection followed by GORevenge and evolutionary selection (up to 150 CpG sites) for the three-class problem (controls *vs.* BCCA *vs.* LYCA) (141 selected CpG sites). Results using the independent set.

Independent Set	1-nn	6-nn	12-nn	Tree	ANN
Total Accuracy	48.46	56.15	55.38	44.62	56.15
Controls Sensitivity	55.56	60.49	54.32	48.15	67.90
BCCA Sensitivity	23.08	53.85	38.46	38.46	53.58
LYCA Sensitivity	41.67	47.22	63.89	38.89	30.56

**Table 11:** Pre-selection followed by evolutionary selection (up to 400 CpG sites) for the three-class problem (controls *vs.* BCCA *vs.* LYCA) (373 selected CpG sites). Results using the independent set.

1-nn	6-nn	12-nn	Tree	ANN	1-nn
Total Accuracy	59.23	58.46	55.38	45.38	69.23
Controls Sensitivity	55.56	53.09	45.68	54.32	80.25
BCCA Sensitivity	61.54	46.15	46.15	61.54	84.62
LYCA Sensitivity	66.67	75.00	80.56	19.44	38.89

Results are reported and commented beneath, firstly on the basis of the performance obtained using resampling on the training set. Then, results are discussed and conclusions are made on the basis of performance we obtained when models constructed using the training set were applied to the totally unknown testing set, used as an independent set. Lists of CpG sites selected for the three-class problem (controls *vs.* BCCA *vs.* LYCA) were finally evaluated and compared in terms of their biological content (GO terms and KEGG pathways).

### 3.1. Performance Obtained Using Resampling

Results show that the provided subsets of features corresponding to equal in number CpG sites achieved moderate or high accuracies and class sensitivity measurements when the training set was solely used along with resampling. This was found both within the evolutionary selection process when applied (reported measurements for the embedded 12-nn classifier are shown in Tables S1–S4,S8,S9 (Column 2), and additionally when features subsets were evaluated by the classifiers on a leave-one-out strategy using the training set (Tables S1–S9, Columns 3–7). Almost all performance measurements were found better that 50%, however, some outliers (performing even down to 35%) existed for the case of the weakest classifiers. The classifiers perform a lot better in the two-class task BCCA *vs.* LYCA, showing that this is an easier distinguishing task than that of distinguishing control from a case (either cancer type), or distinguishing controls from the two cancer types simultaneously. The most well performing classifier overall seems to be the ANN, with the 12-nn classifier following in most classification tasks (expected since it comprises the classifier embedded into the evolutionary feature selection process when applied). For the three-class problem controls *vs.* BCCA *vs.* LYCA the best performing ANN classifier, in particular, achieved performance measurements all in the range of 68% up to 91.4%.The rates achieved for the task BCCA *vs.* LYCA by ANN, the best among all rates achieved for the two-class tasks, are equally to 100% (for all evaluation metrics).

It is encouraging that the different learning algorithms evaluated in the current study were all found to perform adequately well (most of the times far more than 50% for the two-class problems, and far more than 33% for the three-class problem corresponding to random classifiers), when evaluated on a resampling basis using the training set. It should be noted that two more learning algorithms were added to the *k*-nn weighted classifier, which was used internally by the evolutionary feature selection, when selected features and classifiers were evaluated using resampling. Thus, any doubts for a bias introduced by the use of an embedded classifier into the feature selection module could be eliminated.

**Figure 1:**
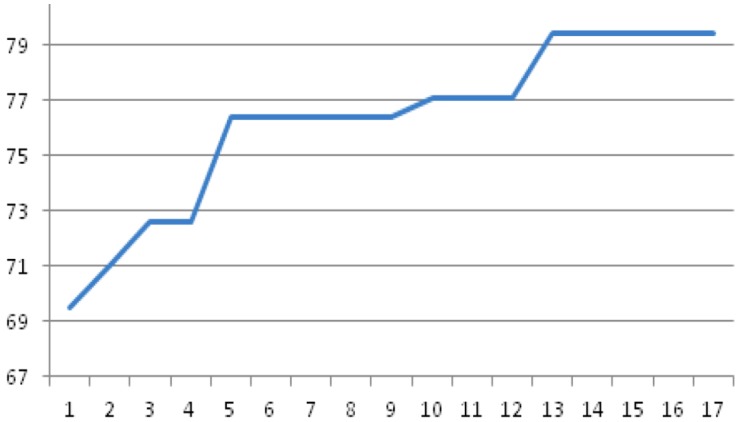
Total accuracy (%) of the embedded 12-nn classifier during GA evolution (three-fold cross-validation) for the three-class problem controls *vs.* BCCA *vs.* LYCA (% accuracy *vs.* number of generations completed).

The evolutionary feature selection method itself performed well when applied: it lowers the dimensionality of the features and selected features perform well based on resampling using the training set (Tables S1–S4,S8,S9). This is due to the fact that the genetic algorithm could screen effectively the complete solution space given the initial population used. Actually, the setting of 500 chromosomes in the initial pool of candidate solutions (each corresponding to 150 or 400 features maximum) ensures that all features (out of 5719 or 352 features submitted to GA depending on the case) could be handled as candidate features for the final solution. GA showed a quick convergence even the low *P_c_* (=0.5) value. The convergence of GA within feature selection for the three-class problem controls *vs.* BCCA *vs.* LYCA is shown in Figure 1: the GA reaches the maximum performance for the embedded classifier already in the 13th generation (out of 50 generations).

On the other side, GORevenge as a feature selection method provides features that perform adequately well when fed to classifiers and tested using resampling on training set. We stress here anyway, that the comparative assessment of the different feature selection scenarios, using results based on resampling on training set, is not the goal of our work (hence results are presented as supplementary). However, we attempt to identify critical performance aspects of the decision mechanism, with respect to the topological structure of the given dataset, on the basis of results obtained using the independent testing set in the following subsection.

### 3.2. Performance Obtained Using the Independent Set—Functional Analysis—General Discussion

**Figure 2:**
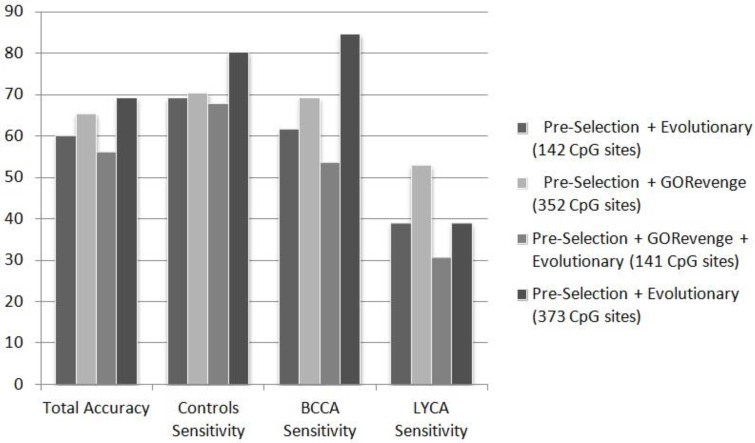
Performance (%) of ANN in the totally unknown—independent testing set in the three-class problem (controls *vs.* BCCA *vs.* LYCA), fed by CpG sites subsets selected by the selection schemes applied.

Regarding the application of the classifiers constructed by the training set and evaluated on the independent testing set, performance is lower than the performance obtained using resampling on the training set. This is a finding in conformance with our expectations and is shown in detail in Table 2, Table 3, Table 4, Table 5, Table 6, Table 7, Table 8, Table 9, Table 10 and Table 11 (comparing to Tables S1–S9) where results obtained using all classification models for all feature selection scenarios and all classification tasks are reported. The results show that the ANN classifier is again here the best-performing classifier overall and probably its superiority is due to its high capability of capturing non-linear effects within the input set. Based on that we attempt to evaluate the feature selection scenarios followed using the performance that the ANN classifier could achieve in the independent testing set. Figure 2 summarizes the results of the ANN classifier for each of the feature selection scenarios for the three-class problem controls *vs.* BCCA *vs.* LYCA. Perusing the epidemiological dataset as a three-class problem represents not only legitimacy, from the data-mining perspective, but also a plausible investigative strategy to be evaluated for its practical performance in real-life problem. The diversity of epidemiological phenotypes that should be inquired from blood tissue screening is immense, and there is a great need for a rapid, standard framework, for initial analysis, prior to more exhaustive investigations. Most of the accuracy or class sensitivity measurements in the three-class problem are greater than 50% far beyond the 33% random choice threshold, however the greater drawback is the sensitivity of B-cell lyphoma class, which samples are mislabeled as controls or breast cancer cases.

**Figure 3:**
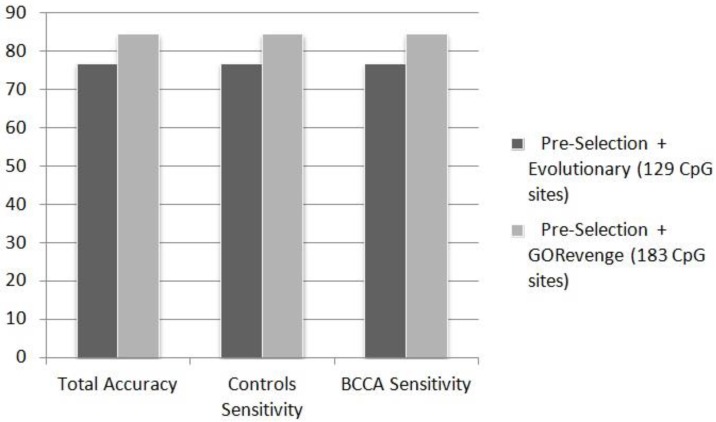
Performance (%) of ANN in the totally unknown—independent testing set in the two-class problem (controls *vs.* BCCA), fed by CpG sites subsets selected by the selection schemes.

From the phenotypic point of view the B-cell lymphoma class, represents an extremely broad family of various lymphatic cancers with diverse molecular mechanism and probably etiology. Given the utilization of 46 out of the 82 samples describing the B-cell lymphoma category, this number is actually meager in order to effectively address ranges of the phenotypic versatility of this category. The subset of CpG sites selected when evolutionary selection was applied to the statistically pre-selected CpG sites, includes a total of 373 CpG sites. This subset is the one to feed the best performing ANN classifier in terms of total accuracy, controls class sensitivity, and breast cancer class sensitivity. However, it does not perform well regarding the B-cell lymphoma class sensitivity (38.89%), for the reasons mentioned above. Regarding this measurement, the ANN fed by the 352 CpG sites proposed by selection based solely to GORevenge showed superior performance. This subset of CpG sites seems to feed an ANN with a balanced performance in terms of sensitivity across all three classes (70.37%, 69.23% and 52.78% for the classes of controls, breast cancer cases and B-cell lymphoma cases). It is worth noting that this subset has been derived solely by using a functional analysis approach, without embedding classification mechanisms within the selection of CpG sites. The subsets of CpG sites proposed either by the evolutionary selection (combined with a threshold=400 for the number of CpG returned) or by GORevenge followed by evolutionary selection seem to perform worse. Figure 3 shows that the 183 CpG sites selected by GORevenge (accuracy: 84.62%, controls sensitivity: 84.62%, cases sensitivity: 84.62%) outperform the 129 CpG sites provided by the evolutionary selection (accuracy: 76.92%, controls sensitivity: 76.92%, cases sensitivity: 76.92%) for the two-class problem controls *vs.* BCCA.

**Figure 4:**
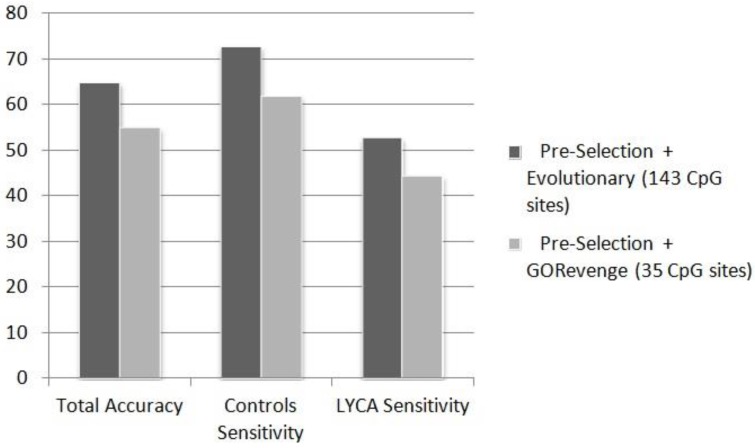
Performance (%) of ANN in the totally unknown-independent testing set in the two-class problem (controls *vs.* LYCA), fed by CpG sites subsets selected by the selection schemes applied.

Figure 4 shows that for the case of the task controls *vs.* LYCA, the subset of 143 CpGs provided by the evolutionary scheme (accuracy: 64.84%, controls sensitivity: 72.73%, cases sensitivity: 52.78%) outperforms the subset of 35 CpG sites, much lower in dimensionality though, provided by GORevenge (accuracy: 54.95%, controls sensitivity: 61.82%, cases sensitivity: 44.44%). The task of recognizing B-cell lymphoma samples from controls prove quite more difficult than discriminating breast cancer samples from controls. This can be, again, attributed to the variety of lymphatic cancers with diverse molecular mechanism compared to the small number of B-cell lymphoma samples exploited to train the constructed classifiers. The two-class task of discriminating breast cancer from B-cell lyphoma in the unknown testing set shows that this task is of moderate complexity and classifiers provide an exceptional performance, almost equal to 100% (Table 6). This is very important as regards the practical utility of the use of this signature for diagnostic purposes, because it manages to mark off the fundamentally different, underlying, molecular underpinnings of these two cancer pathologies, confirming the profoundly different histological nature which, indeed, sets wide apart breast from hematological neoplastic pathologies.

The fact that the performances of the evolutionary selection scheme and GORevenge-based selection are close suggests that the fusion of these orthogonal methodologies might be a promising avenue, in order that one derives biomarkers sets, with both a high classification performance and a biological interpretive value. Experiments not reported here showed that cluster analysis could not provide a visually satisfying separation of the various samples classes (See Figure S1 for clustering controls, BCCA, and LYCA samples using the 352 CpG sites selected by GORevenge). This can be considered an asset for the more sophisticated artificial intelligence methods applied here, that can capture more complex relations.Another approach that we plan to adopt in the near future towards improving the classification performances is the incorporation of bagging schemes, through which we will endeavor to integrate different top-performing classifiers.

Several subsets of CpG sites were provided here by the selection schemes applied that can be used to distinguish (one or two) cancer types from control samples, or one cancer type from another.The subsets of 373 and 352 CpG sites as proposed by the evolutionary feature selection and the GORevenge-based selection to be used for discriminating all three phenotypic classes used here, *i.e.*, controls, breast cancer, and B-cell lymphoma, comprise promising candidates for DNA methylation biomarkers for the two cancer types studied here. These two subsets share a common part of 25 CpG sites whose biological impact is to be studied. Since selected subsets of CpG sites are currently undergoing further biological investigation, in order to highlight putative, causative, theoretical, molecular networks predisposing for disease onset and progression, CpGs sites, corresponding CpG sites are not reported here.

The aim of this study is to examine the suitability, from an epidemiological perspective, of the goal to predict manifestation of two cancer types (breast cancer and B-cell lymphoma) through examination of genome-scalemeasurements of the DNA methylation observed in blood samples of human donors who, at the time of collection, were healthy. In this sense, the derivation of early molecular predictive disease biomarkers, probably years before the macroscopic observation of the two globally-serious epidemiological threats represents a grand challenge. Despite the fact that these diseases possess a diverse phenotypic profile, encompassing very diverse combinations of molecular phenotypes (groups of related genes), the proposed biologically-inspired strategy of selecting biomarkers based on functional analysis yields promising results. This was shown even in the case of the blindfold validation, thus suggesting its expediency as a reliable avenue for robust biomarker discovery.

Towards enlightening the biological content beneath the selected lists of CpG sites for the three-class problem, we have initially performed GO enrichment analysis using the StRAnGER2 algorithm (Available online: www.grissom.gr/stranger2, [43]). Top ten results of the enrichment analysis for the Biological Process category for genes corresponding to the 352 CpG sites derived from the GORevenge-based selection and genes corresponding to the 373 CpGs selected by the evolutionary selectionare presented in Table 12a,b, respectively. In spite of the small overlap between the two CpG lists, both sets of results suggest an epigenetic impact at the level of growth and development-related genes and, more particularly, related to axon guidance and the development of the neuronal system. However, the GORevenge selection captures more biologically-enriched ontological content, which is highlighted by the significantly lower hypergeometric *p*-values and the higher gene enrichments. This finding provides adequate evidence for the plausibility of discovery strategies, which are shoring up in the diligent exploration of the wealth of functional information, regarding the role of genes in specific molecular mechanisms. For instance, GORevenge promoted CpG sites corresponding to 29 out of 345 axon guidance-related genes, whereas the GA derived only 15. Interestingly, the role of axon guidance molecules has been described in tumorigenesis, cancer progression and metastasis [44] In this context, it has been shown that the role of Slit and Robo signaling in various cancer progressions (including breast cancer) is mediated through hypermethylation of their promoters (epigenetic inactivation) [45,46,47]. Remark that the evolutionary selection promoted only Robo4-related CpG sites, whereas the GORevenge selection promoted both Robo and Slit genes (Robo2, Slit3) (see Table S10 for full lists of GO terms and related genes resulting from the two lists of CpG sites). Table 13 reports the genomic distribution of the 352 CpGs sites derived from the GORevenge-based selection in different groups: promoter, body, 3′UTR, and intergenic. It is shown that the majority of selected CpG sites are located in the promoter region

Furthermore, the genes related to selected CpG sites were mapped to canonical pathways by employing KEGG Pathway enrichment analysis using StRAnGER2. Results presented in Table 14 clearly show that the CpG promoted by GORevenge are strongly mapped to well-established cancer-related pathways, in contrast to the evolutionary selection, which yielded poor enrichments and not obviously related to cancer molecular phenotypes. For instance, deregulated Hippo signaling is frequently observed in human cancers as its deregulation leads to a concurrent combination of uncontrolled cellular proliferation and inhibition of apoptosis [48]. In addition, the Wnt and TGFb signaling pathways have been shown to cross-talk with Hippo [48,49] and their signaling is deregulated in many human cancers [50,51]. Lastly, the PI3K-Akt signaling pathway has been shown to be inhibited by Robo1 signaling [46] with a positive impact in breast cancer survival outcomes (see Table S11 in Supplementary Material for lists of KEGG pathways and related genes resulting from the two lists of CpG sites).

**Table 12:** Top enriched GO terms (analysis using the biological process category) derived by genes corresponding to CpG sites selected by GoRevenge-based selection (**a**) and genes corresponding to CpG sites selected by evolutionary selection (**b**). (**a**) Using GORevenge results; and (**b**) using evolutionary selection results.

	GO ID	GO Description	*p*-Value	Enrichment
(**a**)				
1	GO:0048709	oligodendrocyte differentiation	5.52 × 10^−13^	9/21
2	GO:0044281	small molecule metabolic process	1.50 × 10^−12^	66/1530
3	GO:0007411	axon guidance	1.92 × 10^−12^	29/345
4	GO:0046777	protein amino acid autophosphorylation	1.93 × 10^−12^	21/171
5	GO:0051216	cartilage development	3.58 × 10^−12^	14/90
6	GO:0030900	forebrain development	3.86 × 10^−12^	15/87
7	GO:0009790	embryo development	3.97 × 10^−12^	24/154
8	GO:0007420	brain development	4.82 × 10^−12^	27/225
9	GO:0001701	in utero embryonic development	5.75 × 10^−12^	36/256
10	GO:0048011	nerve growth factor receptor signaling pathway	5.89 × 10^−12^	36/286
(**b**)				
1	GO:0033603	positive regulation of dopamine secretion	3.44 × 10^−7^	3/6
2	GO:0016458	gene silencing	7.95 × 10^−7^	3/7
3	GO:0007411	axon guidance	7.50 × 10^−6^	15/345
4	GO:0035249	synaptic transmission, glutamatergic	8.03 × 10^−6^	4/23
5	GO:0045662	negative regulation of myoblast differentiation	1.07 × 10^−5^	3/12
6	GO:0030900	forebrain development	1.29 × 10^−5^	7/87
7	GO:0043523	regulation of neuron apoptosis	1.85 × 10^−5^	4/27
8	GO:0001501	skeletal system development	2.14 × 10^−5^	9/152
9	GO:0031069	hair follicle morphogenesis	2.23 × 10^−5^	4/28
10	GO:0048663	neuron fate commitment	2.66 × 10^−5^	4/29

**Table 13:** Genomic distribution of 352 CpGs derived from the GORevenge-based selection, classified in different groups: promoter, body, 3′UTR, and intergenic.

CpG Location	CpGs	Subgroup	CpGs
Promoter	186	TSS200	41
		TSS1500	82
		5′UTR	36
		1stExon	27
Body	147	-	-
3′UTR	19	-	-
Intergenic	-	-	-

**Table 14:** Enriched KEGG pathways derived by genes corresponding to CpG sites selected by GoRevenge-based selection (**a**) and genes corresponding to CpG sites selected by evolutionary selection (**b**). (**a**) Using GoRevenge results; and (**b**) using evolutionary selection results.

	Pathway ID	Pathway Description	*p*-Value	Enrichment
(**a**)				
1	hsa05205	Proteoglycans in cancer	1.37 × 10^−12^	24/222
2	hsa05215	Prostate cancer	2.58 × 10^−12^	17/88
3	hsa04390	Hippo signaling pathway	5.86 × 10^−12^	20/154
4	hsa04910	Insulin signaling pathway	9.91 × 10^−12^	20/136
5	hsa05200	Pathways in cancer	2.39 × 10^−11^	44/326
6	hsa05166	HTLV-I infection	3.52 × 10^−11^	25/263
7	hsa04020	Calcium signaling pathway	4.52 × 10^−11^	25/181
8	hsa04916	Melanogenesis	5.73 × 10^−11^	15/99
9	hsa04010	MAPK signaling pathway	6.98 × 10^−11^	27/257
10	hsa04722	Neurotrophin signaling pathway	1.18 × 10^−10^	16/118
11	hsa04151	PI3K-Akt signaling pathway	1.22 × 10^−10^	27/341
12	hsa04310	Wnt signaling pathway	2.82 × 10^−10^	17/143
13	hsa05217	Basal cell carcinoma	3.93 × 10^−10^	11/55
14	hsa04510	Focal adhesion	3.97 × 10^−10^	20/204
15	hsa04350	TGF-beta signaling pathway	4.18 × 10^−10^	13/81
16	hsa05202	Transcriptional misregulation in cancer	4.78 × 10^−10^	18/165
17	hsa00053	Ascorbate and aldarate metabolism	7.52 × 10^−10^	8/26
18	hsa05030	Cocaine addiction	2.11 × 10^−9^	10/50
19	hsa00500	Starch and sucrose metabolism	3.30 × 10^−9^	10/52
(**b**)				
1	hsa04260	Cardiac muscle contraction	9.81 × 10^−5^	6/76
2	hsa04974	Protein digestion and absorption	2.29 × 10^−4^	6/87
3	hsa05410	Hypertrophic cardiomyopathy (HCM)	1.28 × 10^−3^	5/85
4	hsa05414	Dilated cardiomyopathy	1.72 × 10^−3^	5/90
5	hsa00061	Fatty acid biosynthesis	2.86 × 10^−3^	1/6
6	hsa05412	Arrhythmogenic right ventricular cardiomyopathy (ARVC)	3.70 × 10^−3^	4/73
7	hsa00460	Cyanoamino acid metabolism	3.97 × 10^−3^	1/7
8	hsa04961	Endocrine and other factor-regulated calcium reabsorption	4.98 × 10^−3^	3/49
9	hsa05030	Cocaine addiction	5.36 × 10^−3^	3/50
10	hsa04512	ECM-receptor interaction	7.41 × 10^−3^	4/86
11	hsa04730	Long-term depression	0.010	3/60

Overall, the results of the enrichment analysis clearly show that the semantics-driven selection by GORevenge promotes CpG sites with enriched biological content, while it preserves the accuracy of the classification. Whereas this approach is limited by the depth of ontological annotation, it enables the derivation of more relevant biomarkers with respect to underlying molecular phenotypes. The performance of this methodology is expected to be constantly improving by the expansion of ontological gene annotations. Thus, the proposed methodology may provide a tool for more effective, semantics-driven targeting of molecular candidates for clinical validation, bridging the gap between the genome-scale studies on large cohorts and the smaller, more focused projects for specific clinical applications (ex. subtyping cancers). In this scope, the proposed methodology could also enhance the exploitation of large publicly available databases (The Cancer Genome Atlas, the Encyclopedia of DNA Elements Consortium and the NIH Roadmap Epigenomics Mapping Consortium) which comprise, in addition to methylation data, gene expression and pathway data.These publicly-available data have only partially been exploited and the application of advanced mining techniques is required for their adaptation to answer more specific questions, in regard to diagnostic and prognostic applications [52].

## 4. Conclusions

An intelligent computational framework was applied to DNA methylation profiling data from an Italian epidemiological cohort comprising breast cancer, B-cell lymphoma and control samples. DNA methylation data, extracted by Illumina’sInfinium Human Methylation 450K Bead Chip, was used to select from an initial set of ~480,000 genome-scale methylation measurements, subsets of CpG sites that correspond to epigenetic biomarkers and show pre-disposition to the particular cancer phenotypes studied here. The framework applied included an evolutionary feature selection scheme, a novel selection scheme based on the semantic exploration in the GO tree, and a suite of classifiers appropriately evaluated on the basis of available data. Results showed that the subsets of CpG sites, delivered by the feature selection schemes, can provide encouraging classification performance measurements obtained both on resampling and testing to an unknown set. The biologically-inspired methodology proposed here of selecting biomarkers yielded promising results both for the various classification tasks undertaken and the biological content delivered, thus comprising another strategy for the derivation of biomarkers from molecular data of various kinds.

## Supplementary Files

Supplementary File 1
