# Supplementary material for: Cancer Biomarkers from Genome-Scale DNA Methylation: Comparison of Evolutionary and Semantic Analysis Methods
Source: Microarrays (Basel). 2015 Nov 27;4(4):647–70. doi: 10.3390/microarrays4040647 (PMC4996413; doi:10.3390/microarrays4040647)
Supplement: Supplementary File 1 [file microarrays-04-00647-s001.zip › microarrays-99114 Tables S1-S9 and Figure S1.pdf]

Supplementary Information

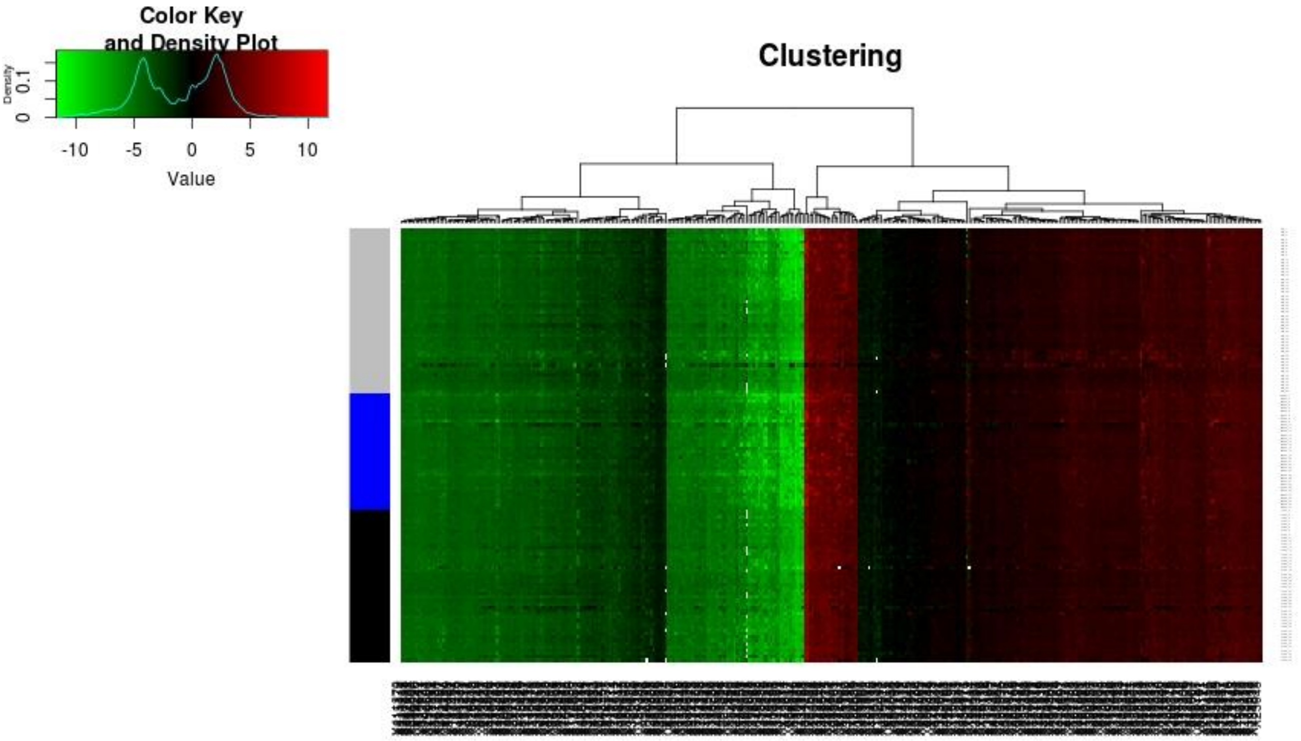

Figure S1. Cluster Analysis of Controls (gray), BCC A (blue) and LYCA (black) samples in the training set using the 352 CpG sites selected by GORevenge.

Table S1. Pre-Selection followed by Evolutionary Selection (up to 150 CpG sites) for the three-class problem (Controls *vs.* BCCA *vs.* LYCA) (142 selected CpG sites). Results using resampling (3-fold cross-validation for embedded 12-nn, leave-one-out for other classifiers).

Resampling	**Embedded 12-nn**	**1-nn**	**6-nn**	**12-nn**	**Tree**	**ANN**
Total Accuracy	79.39	65.65	67.94	87.02	59.54	87.02
Controls Sensitivity	64.00	62.00	58.00	84.00	48.00	84.00
BCCA Sensitivity	88.57	80.00	80.00	88.57	74.29	88.57
LYCA Sensitivity	89.31	58.70	69.57	89.13	60.87	89.13

Table S2. Pre-Selection followed by Evolutionary Selection (up 150 CpG sites) for the two-class problem (Controls *vs.* BCCA) (129 selected CpG sites). Results using resampling (3-fold cross-validation for embedded 12-nn, leave-one-out for other classifiers).

Resampling	**Embedded 12-nn**	**1-nn**	**6-nn**	**12-nn**	**Tree**	**ANN**
Total Accuracy	94.74	82.46	82.46	84.21	73.68	85.96
Controls Sensitivity	86.36	90.91	81.82	86.36	72.73	81.82
BCCA Sensitivity	100	77.14	82.86	82.86	74.29	88.57

Table S3. Pre-Selection followed by Evolutionary Selection (up 150 CpG sites) for the two-class problem (Controls *vs.* LYCA) (143 selected CpG sites). Results using resampling (3-fold cross-validation for embedded 12-nn, leave-one-out for other classifiers).

Resampling	**Embedded 12-nn**	**1-nn**	**6-nn**	**12-nn**	**Tree**	**ANN**
Total Accuracy	82.56	48.65	64.86	71.62	60.81	78.38
Controls Sensitivity	67.86	42.68	50	53.57	53.57	57.14
BCCA Sensitivity	91.3	52.17	73.91	82.61	65.22	91.3

Table S4. Pre-Selection followed by Evolutionary Selection (up 150 CpG sites) for the two-class problem (BCCA *vs.* LYCA) (146 selected CpG sites). Results using resampling (3-fold cross-validation for embedded 12-nn, leave-one-out for other classifiers).

Resampling	**Embedded 12-nn**	**1-nn**	**6-nn**	**12-nn**	**Tree**	**ANN**
Total Accuracy	100	98.77	97.53	98.77	100	100
BCCA Sensitivity	100	97.14	94.29	97.14	100	100
LYCA Sensitivity	100	100	100	100	100	100

Table S5. Pre-Selection followed by GORevenge for the three-class problem (Controls *vs.* BCCA *vs.* LYCA) (352 selected CpG sites). Results using leave-one-out resampling.

Resampling	**1-nn**	**6-nn**	**12-nn**	**Tree**	**ANN**
Total Accuracy	52.67	56.49	64.89	54.20	78.63
Controls Sensitivity	50.00	46.00	44.00	40.00	70.00
BCCA Sensitivity	60.00	62.86	74.29	57.14	91.43
LYCA Sensitivity	50.00	63.04	80.43	67.39	78.26

Table S6. Pre-Selection followed by GoRevenge for the two-class problem (Controls *vs.* BCCA) (183 selected CpG sites). Results using leave-one-out resampling.

Resampling	**1-nn**	**6-nn**	**12-nn**	**Tree**	**ANN**
Total Accuracy	75.44	84.21	84.21	57.89	91.23
Controls Sensitivity	81.82	81.82	72.73	54.55	86.36
BCCA Sensitivity	71.43	85.71	91.43	60.00	94.29

Table S7. Pre-Selection followed by GoRevenge for the two-class problem (Controls *vs.* LYCA) (35 selected CpG sites). Results using leave-one-out resampling.

Resamping	**1-nn**	**6-nn**	**12-nn**	**Tree**	**ANN**
Total Accuracy	58.11	58.11	63.51	60.81	66.22
Controls Sensitivity	39.29	39.29	35.71	42.86	53.57
LYCA Sensitivity	69.57	69.57	90.43	71.44	73.91

Table S8. Pre-Selection followed by GORevenge and Evolutionary Selection (up to 150 CpG sites) for the three-class problem (Controls *vs.* BCCA *vs.* LYCA) (141 selected CpG sites). Results using resampling (3-fold cross-validation for embedded 12-nn, leave-one-out for other classifiers).

Resampling	**Embedded 12-nn**	**1-nn**	**6-nn**	**12-nn**	**Tree**	**ANN**
Total Accuracy	76.27	56.49	57.25	62.60	48.09	76.34
Controls Sensitivity	66.00	54.00	48.00	46.00	30.00	68.00
BCCA Sensitivity	74.29	57.14	60.00	62.86	68.57	82.86
LYCA Sensitivity	89.13	58.70	65.22	80.43	52.17	88.43

Table S9. Pre-Selection followed by Evolutionary Selection (up to 400 CpG sites) for the three-class problem (Controls *vs.* BCCA *vs.* LYCA) (373 selected CpG sites). Results using resampling (3-fold cross-validation for embedded 12-nn, leave-one-out for other classifiers).

Resampling	**Embedded 12-nn**	**1-nn**	**6-nn**	**12-nn**	**Tree**	**ANN**
Total Accuracy	64.83	64.12	70.23	68.70	54.20	77.86
Controls Sensitivity	40.00	66.00	64.00	52.00	40.00	68.00
BCCA Sensitivity	68.57	62.86	77.14	80.00	62.86	94.29
LYCA Sensitivity	89.13	63.04	71.74	78.26	63.04	76.09
